# Special Issue “Arenaviruses 2020”

**DOI:** 10.3390/v13040703

**Published:** 2021-04-18

**Authors:** Igor S. Lukashevich, Juan Carlos de la Torre

**Affiliations:** 1 Department of Pharmacology and Toxicology, School of Medicine, University of Louisville, Louisville, KY 40202, USA; 2 Center for Predictive Medicine for Biodefense and Emerging Infectious Diseases, University of Louisville, Louisville, KY 40202, USA; 3 Department of Immunology and Microbiology, The Scripps Research Institute, La Jolla, CA 92037, USA

**Keywords:** molecular virology of mammalian arenaviruses, lymphocytic choriomeningitis virus (LCMV), Lassa virus (LASV), Lassa fever, vaccine development

Rodent-borne arenaviruses have been traditionally predominantly associated with certain muroid species from *Mastomys/Praomys* genera (African arenaviruses) or with species that belong to murid subfamily *Cricetidae* (New World arenaviruses). However, the implementation of metagenomics tools during recent years has resulted in the discovery of numerous arenaviruses not only in traditional mammal hosts, but also in snakes and fish. These discoveries led to the creation within the *Arenaviridae* family of three new genera, *Antennavirus*, *Hartmanivirus*, and *Reptarenavirus*, to accommodate arenaviruses discovered in snakes (hartmani-and reptarenaviruses) and fish (antennaviruses). The largest genus, *Mammarenavirus*, also added new viruses isolated from rodents captured in different areas of the Americas, Africa, and Asia [1,2]. Some of these new mammalian arenaviruses can infect humans and further investigations are needed to assess their potential role as human pathogens. There is a growing body of evidence indicating that the high degree of genetic plasticity exhibited by mammarenaviruses can facilitate zoonotic spillover and adaptation to new hosts (e.g., ticks, bats), which could have important implications for the epidemiology of these viruses [3]. 

Eleven papers (nine experimental publications and two review articles) included in this Special Issue describe recent progress in the molecular biology and pathogenesis of mammalian arenaviruses with well-known pathogenic potential for humans, lymphocytic choriomeningitis virus (LCMV), and Lassa virus (LASV). LCMV infection of the mouse is one the most widely used models for the investigation of immunological aspects of acute and persistent viral infections. On the other hand, since 2015, the number of Lassa fever (LF) cases and their case fatality ratio have increased significantly in Nigeria, which has motivated several international organizations (WHO, CEPI, IAVI) to combine their efforts to develop LASV vaccines. Different aspects of LASV-host cell interactions, including modulation of the host innate immune responses, as well as the natural history of LASV infection in non-human primates, and the implications for vaccine development of LASV genetic heterogeneity are discussed in this Special Issue. 

LASV enters the human body via two major gates, respiratory tract (inhalation of contaminated dust) and gastro-intestinal tract (ingestion of contaminated food). In both cases, interaction of LASV with polarized epithelial cells and crossing mucosal barriers drive innate host responses and determines the outcome of the infection. The natural history of LF in cynomolgus macaques exposed to aerosolized LASV (Josiah strain) has been described in the paper presented by Downs et al. After a sub-clinical incubation period, fever and viremia were detected on day 4 post-exposure (DPE 4) and viremia levels picked at DPE 10–13. At early clinical stages (DPE 4–5), LASV glycoprotein-positive activated monocytes were detected, as well as lymphocytopenia, tachycardia, decreased appetite, and urination. Disease progression was associated with high viral load in plasma and tissues, blood alkalosis, elevated levels of markers of liver and kidney damage, leukopenia, anemia, thrombocytopenia, and hypoalbuminemia. All these events resulted in hypovolemic shock. Immune dysregulation observed at early stage included low levels of serum cytokines and poor LASV-specific T cell responses. Subcutaneous infection of cynomolgus monkeys with LASV (Josiah) caused progressed disease with similar clinical manifestations [4]. These results correlate well with predictors of fatal disease in LF patients, where high viremia and viral load in tissues, cytokine/chemokine dysregulation, and defective T cell responses are major driving forces resulting in multi-organ failure and sepsis-like hypovolemic shock. These findings justify early therapeutic intervention aimed at controlling viremia and intravenous volume to prevent hypovolemic shock. 

LF modeling in non-human primates and post-mortem LF histology reports did not find a correlation between LASV load in organs and tissue distribution of functional alpha-dystroglycan (α-DG), a major cellular human receptor for LASV and LCMV. In LASV-infected primates and in fatal cases of human LF, postmortem liver samples contain the highest titers of infectious LASV, while matured hepatocytes do not express functional α-DG. Biosynthesis of α-DG includes complex O-glycosylation involving the glycosyltransferase like-acetylglucosaminyl transferase (LARGE). Only LARGE-modified α-DG is required for LASV binding [5]. A growing body of evidence indicates that LASV and LCMV can enter cells in α-DG-independent manner employing additional receptors. In this issue, Fedeli et al. investigated the role of Axl, a member of phosphatidylserine-binding TAM receptors (Tyro3/Axl/Mer), and hepatocyte growth factor receptor (HGFR) in LASV cell entry. To overcome the limited access to BSL4 high-containment facilities required for the use of live LASV, the authors use a recombinant LCMV where LASV glycoprotein (GP) was substituted for LCMV GP. They documented that in the absence of functional α-DG, both Axl and HGFR mediated LASV cell entry via an unusual micropinocytosis pathway that depends on sodium proton exchangers (NHE) and actin. Notably, interfering with the function of Axl and HGFR inhibited cell entry of rLCMV/LASVGP, suggesting a potential therapeutic strategy targeting a very early stage of LASV infection.

The participation of the lysosomal-associated membrane protein-1 (LAMP-1) as an intracellular receptor required for completion of virus cell entry, is a unique feature of LASV that is not shared by closely related mammarenaviruses [6]. Interestingly, the mammarenavirus Lujo virus (LUJV), identified in 2009 as the causative agent of a cluster of fatal cases of viral hemorrhagic fevers in South Africa and Zambia, uses also a receptor switch mechanism to complete its cell entry process. LUJV binds to its primary receptor, neuropilin-2, at the cell surface and within the late endosome switches to CD63 (tetraspanin) to facilitate the GP-mediated fusion event between viral and cellular membranes required to complete the virus cell entry process [7]. In this issue, Bulow et al. presented results of “lipid mixing” visualization using a single-particle fluorescence assay to track LASV-GP-mediated fusion at endosomal pH and a physiological temperature. In this assay, exposure of GFP-encoding VSV pseudotyped with LASV GP to acidic pH triggered the transition of GP from the pre-fusion to the post-fusion conformation. While acidic pH was sufficient to trigger LASV GP-mediated lipid mixing in the absence of interaction with either α-DG or LAMP-1, binding LAMP-1 increased the kinetics of lipid mixing and facilitated robust fusion at a less acidic pH in line with previously published observations [8]. These findings indicate that LAMP-1 is not absolutely required for virus entry but increases the efficiency of LASV infection promoting fusion in less acidic endosomal compartments. 

The LF case report described by Magassouba et al. is a reminder of the difficulties of recognizing LF, which can contribute to a significant number of undiagnosed cases in endemic areas. An individual presented himself at several health centers of Kissidougou province of Guinea with persistent fever, vomiting, and joint pain. He was repeatedly treated with anti-malaria drugs and died three weeks later after admission to Mamou hospital. Prior to death, the patient started bleeding raising the suspicion of Ebola hemorrhagic fever but testing revealed the presence of LASV RNA in blood. Phylogenetic analysis showed that the virus was related to some Liberian isolates that appeared to emerge around 150 years ago. Extensive timber trade between Guinea and Liberia was suggested as a possible epidemiological cause of LASV emergence in Kissidougou area. 

LASV is highly diverse genetically with up to seven lineages being currently recognized that tend to cluster to certain geographic areas. LASV lineages I-III are present in different regions of Nigeria, whereas the largest lineage IV circulates in Liberia, Guinea, and Sierra Leone. Recently, the new lineages V and VI, were identified in Mali and Ivory Coast and in Nigeria, respectively, and two new sequences from Togo were classified as members of the new lineage VII. In this issue, Forni and Sironi used different bioinformatics approaches to analyze current LASV phylogeny and gain insights about LASV origins and possible association between LASV genetic diversity and severity of LF in endemic areas. They found evidence of east-west and north-south geographic gradients driving LASV genetic diversity. They confirmed the previous finding that LASV originated in Nigeria and migrated westward to reach Ivory Coast, Guinea, Liberia, and Sierra Leone [9]. This migration was associated not only with rodent movements but also linked to colonial human migration, transportation, and trade activities. Interestingly, LASV sequences with largest ancestral contribution, e.g., the prototype Pinneo strain (lineage I), have been rarely detected during the last decades. Meanwhile, lineages II and IV are commonly associated with human LF in Nigeria and non-Nigerian endemic areas, respectively. It seems that the ancestral lineage I was replaced by other lineages with higher finesses in the LASV natural reservoir, or ancestral strains were more commonly associated with mild or non-clinical infections. The role of LASV genetic diversity in the efficiency of rodent-to-human transmission or severity of LF remains to be determined. Analysis of LASV sequences belonging to sub-lineage IIg and linked to an LF outbreak with high fatality rates in Tabara state (Nigeria) identified ancestry sequences possibly associated with protection against fatal disease. However, with limited sampling, these results must be interpreted very cautiously.

In this issue, Ibukun examined the effect of LASV genetic diversity on inter-lineage variations of LASV glycoprotein B- and T-cell epitopes. Alignments of GP sequences from all LASV lineages revealed that LASV GP has a limited degree of amino acid conservation within predicted B- and T-cell epitopes. This observation may complicate the development of a “pan-LASV” vaccine effective against all LASV lineages [10]. Nevertheless, predicted conformational B-epitopes appear to be less affected by LASV diversity, providing the opportunity to design a putative vaccine candidate expressing LASV GP in its pre-fusion configuration to induce broadly protective neutralizing antibodies. However, since natural protection induced by infection with attenuated LASV strains is not associated with humoral responses, this vaccine approach would require extensive pre-clinical studies. 

Defective viral genomes (DVG) described by Jonson et al. in this issue provides a possible strategy to overcome LASV genetic diversity for the development of a universal LASV vaccine. Using the promising LASV vaccine candidate ML29 [11], a reassortant between LASV and the non-pathogenic genetically related Mopeia virus (MOPV), the authors documented that replication of ML29 during acute or persistent infections in cultured cells was associated with the generation of DVG derived from the L RNA. The mismatch between L polymerase (MOPV) and NP (LASV) may promote the production of the abundant truncated L-derived 1.5 kb RNA species contributing to the high immunogenic properties of ML29 via activation of host pattern recognition receptors (RIG-I, TLRs, PKR). In persistently infected ML29 cells, accumulation of the L-derived RNA species was associated with attenuation and homologous (ML29) and heterologous (LASV, MOPV, LCMV) interference. Generation of DVG and interference are classical features of defective interfering particles (DIPs). DIP-enriched ML29 preps were highly attenuated in immunocompetent mice and guinea pigs. These preps did not induce hearing abnormalities in STAT-1^-/-^ mice, a small animal model for hearing loss sequelae commonly observed in LF survivors. A growing body of evidence indicates that DIPs can be attractive tools for the development of a new generation of adjuvants and biologics to target genetically diverse and rapidly evolving viruses.

Progression of LF disease is associated with immunosuppression contributed to high LASV load in blood and tissues and multi-organ functional failure. Mechanisms of host immune modulation by NP and Z proteins of pathogenic and non-pathogenic mammarenaviruses were investigated in the paper by Stott and al. NP is the most abundant viral protein in infected cells and is required for the formation of the virus ribonucleoprotein complex (vRNP), responsible for directing the biosynthetic processes of replication and transcription of the viral genome. In addition, mammarenavirus NP has been shown to be a potent inhibitor of induction of the type I interferon (IFN-I) response by the infected cell. This NP anti-IFN-I activity has been linked to a functional 3′-5′ exoribonuclease domain of the type DEDDh present at the C-terminal region of NP. This domain is highly conserved among mammarenaviruses regardless of their human pathogenic potential. NP has been shown to inhibit IRF-3 phosphorylation and nuclear translocation, as well as activation of NF-kB and PKR. These features were described for pathogenic and non-pathogenic mammarenaviruses, suggesting that NP-mediated innate immune evasion is more related to the establishment or maintenance, or both, of mammarenavirus persistent infections in their rodent natural reservoirs, rather than immunosuppression in infected individuals. Nevertheless, NP-mediated contribution to poor LASV immunity cannot be excluded [12]. Similar to NP, arenavirus Z protein, via its N-terminal domain, affects RIG-I-MAVS signaling resulting in IFN-I suppression. However, this feature of the Z protein was predominantly linked to pathogenic mammarenaviruses, since recombinant Z expressing N-terminal domain of non-pathogenic Pichinde virus failed to suppress IFN-I response [13]. In this issue, Ling et al. extended these studies using LASV isolates from humans and rodents. Notably, while all analyzed LASV Z proteins inhibited human RIG-I function in vitro, this inhibition was slightly stronger for Z proteins from human LASV isolates versus LASV isolated from rodents. Similar to LASV, Z proteins of several LCMV strains were also associated with RIG-I inhibition. It has to be further investigated if Z-mediated inhibition of RIG-I activity is associated with clinical manifestations of disease caused by pathogenic mammarenaviruses.

Strain WE of LCMV causes an LF-like fatal hepatitis in NHPs [14]. This strain is less studied than the commonly used Armstrong (ARM) strain of LCMV that causes subclinical infections in primates. Both strains share 82% and 85% of nucleotide identity for the L and S genomic RNA, respectively. In this issue, Taniguchi et al. developed a reverse genetics system for LCMV-WE to study the untranslated regions (UTRs) of viral genome that are essential for viral replication and transcription. Focusing on the S RNA UTRs, the authors found that internal UTRs (nt 41–77 and 39–60, at the 5′ and 3′ termini, respectively) were linked to pathogenic features of LCMV-WE since mutations and deletions in these regions resulted in attenuated infection in mice. This observation is consistent with previous findings indicating that non-coding intergenic regions of LCMV are involved in virulence and can be rationally targeted to design attenuated vaccines [15]. It should be feasible now to generate chimeric viruses between WE and ARM strains of LCMV to dissect the molecular mechanisms responsible for WE-induced fatal disease in primates.

Finally, the paper presented by Kim et al., documents novel inhibitors of dihydroorotate dehydrogenase (DHODH), a key cellular enzyme in de novo pyrimidine biosynthesis, with potent activity against LASV and Junin virus (JUNV), a New World mammarenavirus and causative agent of Argentine HF. This inhibition was IFN-independent and targeted viral RNA synthesis suggesting that pyrimidine depletion is a major mechanism of action mediated by these inhibitors. Experiments with LCMV-infected mice treated with some of these inhibitors provided moderate success. Combination treatment targeting the pyrimidine salvage pathway has been proposed as a strategy to enhance the antiviral effect of promising DHODH inhibitors.

Co-editors of this Special Issue deeply acknowledge of all authors for their contributions and dedication to accomplish this project in the challenging COVID-19 pandemic time. 
